# Cancer Risks Associated with Arsenic in Drinking Water

**DOI:** 10.1289/ehp.9927

**Published:** 2007-07

**Authors:** How-Ran Guo

**Affiliations:** Department of Environmental and Occupational Health Medical College National Cheng Kung University Tainan, Taiwan E-mail: hrguo@mail.ncku.edu.tw

Arsenic in drinking water is a worldwide problem, and studies in southwest Taiwan (Chen et al. 1985, 1988; Tseng 1977; Tseng et al. 1968; Wu et al. 1989) have been the bases of many cancer risk assessments. In a recent reanalysis of the data, Lamm et al. (2006) found “township” a confounder. Specifically, of six townships, only three (2, 4, and 6) showed positive dose–response relationships with arsenic exposure, and three others (0, 3, and 5) demonstrated cancer risks independent of arsenic exposure. The authors speculated that the confounding was related to black-foot disease (BFD), a peripheral vascular disease associated with arsenic ingestion (Ch’i and Blackwell 1968; Tseng 1977), but they did not know the identities of the individual townships.

On the basis of data collected in previous studies (Brown et al. 1997, 2000; Kuo 1968), townships 0, 2, 3, 4, 5, and 6 (Lamm et al. 2006; National Research Council 1999) can be identified as I-chu, Pu-tai, Hsieh-chia, Yen-shui, Pei-men, and Hsia-ying. With the highest prevalence of BFD in the country, I-chu, Pu-tai, Hsieh-chia, and Pei-men are generally referred as the “BFD-endemic area.” Therefore, the “township factor” might be indeed related to BFD, because all three of the townships affected by this factor were in the endemic area. Pu-tai was the only BFD-endemic township not affected by the factor, and two of the five villages included in the analysis are in the northern part of the township, where the prevalence was low; this might be why it appeared to be a nonendemic township.

Lamm et al. (2006) further speculated that the township factor was a reflection of a selection bias because the water sampling was focused on villages with high BFD prevalence. Although the sampling was related to BFD cases, the chance of a bias occurring in the selection of villages by Wu et al. (1989) was small because they included almost all of the villages covered in the survey by Kuo (1968) in the six townships.

Lamm et al. (2006) claimed that their finding of a threshold-like model indicating no increase of bladder cancer with exposure levels < 150 μg/L was consistent with results from toxicologic studies and other epidemiologic data from the United States, Argentina, and northeastern Taiwan. Actually, it is also consistent with studies on bladder cancers covering the whole of Taiwan (Guo et al. 1997), southwest Taiwan only (Guo 1999; Guo and Tseng 2000), and another reanalysis of the same data (Morales et al. 2000; Stöhrer 2001). Furthermore, their results are consistent with studies on lung (Guo 2004) and skin (Guo et al. 1998, 2001) cancers. Whereas Lamm et al. (2006) stated that low-dose villages showed a negative dose–response curve for bladder and lung cancers, they reported a positive slope (1.275) in their Figure 2; this is likely to be an error. In previous studies, exposures between the detection limit (0.001 ppm) and 0.01 ppm had a significant negative effect on transitional cell cancer of the kidney and on skin cancer in both sexes; the negative effect on bladder cancer was also significant in women, but not in men (Guo et al. 1994, 1998).

Even if the dose–response relationship fits a threshold-like model, using the median arsenic level in each village as the exposure indicator might not generate accurate risk estimates because villages with similar median arsenic levels can have very different distributions of exposures. For example, villages 0-G and 3–5 had very close median arsenic levels, 0.030 and 0.032 ppm, respectively (Lamm et al. 2006); if there was a threshold at 0.150 ppm, as proposed by Lamm et al., one would expected an increased risk in village 0-G, where the residents had exposure levels up to 0.770 ppm, but not in village 3–5, where all residents were assigned the exposure level of 0.032 ppm. In fact, previous studies on urinary cancers in Taiwan suggested an inflection point > 0.32 ppm (Guo 1999; Guo et al. 1994, 1997). If 0.32 ppm is adopted as the cutoff, villages 0-G, 0-E, 0-I, and 3-Q would be placed in the “low-dose villages” group, although they should have increased risks because some of the residents had exposures above the threshold. These four villages were in the township group “0, 3, and 5,” which Lamm et al. regarded as being affected by the township factor, but no villages in the other township group (townships 2, 4, and 6) had this problem. Therefore, misclassifications might also contribute to the township factor; a different choice of exposure indicator may help clarify the uncertainties.
